# Editorial: Psychological Models for Personalized Human-Computer Interaction (HCI)

**DOI:** 10.3389/fpsyg.2021.673092

**Published:** 2021-04-06

**Authors:** Bruce Ferwerda, Li Chen, Marko Tkalčič

**Affiliations:** ^1^Department of Computer Science and Informatics, School of Engineering, Jönköping University, Jönköping, Sweden; ^2^Department of Computer Science, Hong Kong Baptist University, Hong Kong, Hong Kong; ^3^HICUP Lab, Faculty of Mathematics, Natural Sciences and Information Technologies, University of Primorska, Koper, Slovenia

**Keywords:** personalization, user modeling, human computer interaction, psychological theories and models, user characteristic

## 1. Introduction

The behavior of users in the digital world, such as online shopping or social media activity, is increasingly supported by personalized systems, such as recommender systems (Ricci et al., 2015) and personalized learning. Early work on personalized systems was mainly data-driven, based on behavioral data, such as ratings, likes, and purchases (e.g., Bell et al., 2007). Although these systems are useful for both users and service providers, the main downside is the limited interpretability and explainability of the data. Such limitations in both interpretability and explainability translate in using data without understanding the root-cause of behaviors. Recent work has thus started to adopt a more theory-driven approach by including psychological theories and models to improve personalized systems (see for an overview; Graus and Ferwerda, 2019). These systems take advantage of psychological theories/models, such as emotions (Tkalčič et al., 2013b; Tkalčič and Ferwerda, 2018), personality (Ferwerda et al., 2017; Wu et al., 2018), skills (Ferwerda and Graus, 2018), and culture (Schedl et al., 2017) to explain and predict behaviors of users. This allows for a deeper understanding of users' behavior, preferences, and needs, which in turn also lead to more generalizable results.

Moreover, digital behavior has also been used to infer user traits and characteristics. For example, social media activities have been used to predict personality traits (Skowron et al., 2016) and intelligence, whereas the field of affective computing has been active in devising methodologies for inferring emotional states from digital signals (Tkalčič et al., 2013a).

## 2. Research Topic Content

In view of this situation, this Research Topic aimed at collecting state-of-the-art research that supports personalized services with psychological theories/models. In particular, we encouraged the authors to submit original research articles, case studies, reviews, theoretical and critical perspectives, and viewpoint articles on the following topics: (i) *Psychological theories/models that explain online behavior* (e.g., personality, emotions, cognitive biases and illusions, learning styles, emotional contagion in group settings), (ii) *Psychological theories/models to personalize digital interactions* (e.g., in user interfaces, recommendations, social robots and chat-bots, e-learning), and (iii) *Prediction of psychological models drawing data from digital behavior information resources* (e.g., social media, e-commerce, physical activities, online learning, group scenarios).

Within this collection we accepted 13 works. In total there were 11 original research articles, one brief research report and one perspective article. The authors affiliation countries were diverse, including Europe (Germany, Italy, Poland, Austria, Norway, and Sweden), North America (USA and Canada), and Asia (Pakistan, Japan, Malaysia, China, and South Korea).

The topics cover (i) *user characteristics* [technology acceptance (Pan), attachment styles (Sessa et al.), cognitive styles (Steichen and Fu; Schürmann and Beckerle), jealousy (Nordmo et al.), psychopatology (Sorokowski et al.), motivation (Huifeng and Ha; Hulaj et al.), needs (Hulaj et al.), personality (Xu and Ye; Abbasi et al.), and emotion (Cecconi et al.)], (ii) *inference* [from eye gaze (Steichen and Fu) and from social media (Cecconi et al.)], and (iii) *personalization* [survey (Neumayr and Augstein) and human-agent interaction (Schürmann and Beckerle)] in a (iv) *wide range of scenarios* [learning (Pan), communication (Sessa et al.; Miyamoto et al.), information visualization (Steichen and Fu), human-robot interaction (Nordmo et al.; Schürmann and Beckerle), consumer termination (Huifeng and Ha), video games (Hulaj et al.; Abbasi et al.), live streaming (Xu and Ye), and social media (Cecconi et al.)].

In this work, Pan  explores how technology acceptance and self-efficacy contribute to the attitude toward technology-based self-directed learning. His results indicate a high relationship between these factors.

Sessa et al. explore how the attachment style influences the reaction in case of displeasing messages. Their results indicate that the communication styles of frankness and mitigation are related to attachment styles.

The psychological acceptability of utterances has been shown to be influenced by the social distance in the study conducted by Miyamoto et al..

The study conducted by Abbasi et al.  was researching the relationship between personality and video games engagement. The results they obtained suggest that openness to experience, extraversion, agreeableness, and conscientiousness positively predict consumer engagement in electronic sports games.

Xu and Ye  aimed at understanding the personality traits and the motivations of active live streaming viewers as well as their user behaviors in the general population in China. Their results indicate that extraversion was negatively associated with live streaming use, while openness was positively associated.

The emotion of Schadenfreude, pleasure at another's misfortune, has been investigated by Cecconi et al. , who found that, in an corpus of social media posts in italian, a set of hashtags (e.g., #Glistabene, #Benglista = hedeservedit) are strong predictors of shadenfreude.

Schürmann and Beckerle  propose a framework for designing cognitive models for a given research question. The framework consists of five external and internal aspects related to the modeling process: research question, level of analysis, modeling paradigms, computational properties, and iterative model development.

Steichen and Fu found that a user's cognitive style can be inferred from the user's eye gaze while using an information visualization system.

Neumayr and Augstein  present a systematic survey of personalized collaborative systems.

Nordmo et al. investigated the intimate relationship between humans and robots. They found that females expect to feel more jealousy if their partner got a sex robot, rather than a platonic love robot.

Hulaj et al.  carried out a study investigating factors that influence dthe performance in video games in terms of matchmakin rating (MMR). They found that the perceived competence and autonomy were the only significant predictors of MMR performance beyond matches played.

Huifeng and Ha  investigated what influences the termination of a customer relationship and found several factors: upkeep, time, benefits, personal loss, and motivation.

A research on the relationship between psychopatological personal traits and online hate behavior was conducted by Sorokowski et al. . Their results show that high scores in Psychopathy subscale are significant predictors of posting hating comments online.

## Author Contributions

BF, LC, and MT wrote sections of the manuscript. All authors contributed to manuscript revision, read, and approved the submitted version.

## Conflict of Interest

The authors declare that the research was conducted in the absence of any commercial or financial relationships that could be construed as a potential conflict of interest.
